# Complete mitochondrial genomes of Baikal endemic coregonids: omul and lacustrine whitefish (Salmonidae: *Coregonus* sp.)

**DOI:** 10.1080/23802359.2019.1703565

**Published:** 2020-01-10

**Authors:** Veronika Teterina, Lyubov Sukhanova, Vasiliy Smirnov, Natalya Smirnova, Sergei Kirilchik, Yulia Sapozhnikova, Olga Glizina, Vera Yakhnenko, Marina Tyagun, Tuyana Sidorova

**Affiliations:** a Limnological Institute Siberian Branch of the Russian Academy of Sciences, Irkutsk, Russia;; b Baikal Museum of Irkutsk Scientific Center of the Siberian Branch of the Russian Academy of Sciences, Listvyanka, Russia

**Keywords:** Baikal omul, *Coregonus migratorius*, Baikal whitefish, *Coregonus baicalensis*, mtDNA, mitochondrial genome

## Abstract

Coregonid fishes are among the most successful groups in the subarctic, boreal, and subalpine fresh waters of the northern hemisphere. Limnetic–benthic sympatric species-pairs from two different evolutionary lineages, the North American lake whitefish (*Coregonus clupeaformis* species complex), and the European whitefish (*Coregonus lavaretus* species complex), are becoming the subject of close attention to explore the role of natural selection during the ecological speciation. Baikal endemic coregonids, limnetic omul (*Coregonus migratorius*), and benthic lacustrine whitefish (*Coregonus baicalensis*) are the only representatives of another unique lineage that has not left the lake since the divergence from the two above. Due to Pleistocene oscillations sympatric limnetic–benthic divergence has been replicated here many times within the same water body over a long geological period in contrast to both Europe and America where sympatric species-pairs are the results of post-glacial secondary-contacts between glacial isolates during the Late Pleistocene on the territory of each continent. Mitochondrial genomes encode genes that are essential for respiration and metabolism. Data on complete mitogenomes of Baikal endemic coregonids provided here will complement ongoing investigations on energy metabolism as the main biological function involved in the divergence between limnetic and benthic whitefish.

In contrast to numerous studies that focus on the genomic basis of adaptive phenotypic divergence, the role of gene expression during speciation has been much less investigated and, consequently, less understood (Rougeux et al. 2019).

‘Nonmodel’ species studied in their ecological context, such as whitefish, play an increasingly important role in ecological genomics (Bernatchez et al. 2010; Rougeux et al. 2019). Transcriptomic studies of these fish show that energy metabolism is the main biological function involved in the divergence between limnetic and benthic whitefish (Trudel et al. 2001; Bernatchez et al. 2010; Rougeux et al. 2019). There is mounting evidence that selection has been acting more strongly on limnetic than benthic whitefish and special attention is given to metabolic genes associated with the mitochondrion machinery (Derome et al. 2006; St-Cyr et al. 2008).

Lake Baikal is one more unique place to study genetic and phenotypic divergence among sympatric whitefish ecotypes (Bychenko et al. 2014). Obviously, in Baikal, in comparison with North American and European lakes, selection has been acting on limnetic ecotype even more strongly. Complete reproductive isolation of ecotypes by spawning time (autumn/winter) and place (rivers/lake shoals) (Skryabin 1969) as well as pronounced intraspecific phenotypic structure, of limnetic ecotype, testify it (Smirnov 1992).

To explore adaptation to the deepest oligotrophic lake with a highly superstructured vast pelagic zone (Shimaraev et al. 1994) and to trace parallelisms between sympatric pairs through the continents, we present the first complete mitogenomes for Baikal endemic coregonids: limnetic – omul *C. migratorius* and benthic – lacustrine whitefish *Coregonus baicalensis*.

All the samples were collected directly in Lake Baikal and its basin during fish spawning migrations. Total genomic DNA was isolated from fin clips collected from three specimens for each species. The exact collection sites for each sample placed in the GenBank were as follows:

1. *C. migratorius* MN394787 – Barguzin River (Baikal’s tributary) – 53°30′11 N; 109°21′31 E
2. *C. migratorius* MN394788 – Barguzin River (Baikal’s tributary) – 53°30′11 N; 109°21′31 E
3. *C. migratorius* MN394789 – Kulinda Lake (Lake Baikal basin) – 56°07′13 N; 110°28′03 E
4. *C. baicalensis* MN394784 – Chivyrkuy Bay (Lake Baikal) – 53°45′35 N; 109°04′21 E
5. C. baicalensis MN394785 – Delta of the Selenga River (Lake Baikal) – 52°27′01 N; 106o39′45 E
6. *C. baicalensis* MN394786 – Maloye More Strait (Lake Baikal) – 53°03′30 N; 106°51′38 E

Voucher material from the list above was retained at Baikal Museum of ISC SB RAS, Listvyanka, Russia under Accession numbers as follows: 1. Bar376; 2. Bar377; 3. Kul52; 4. Chiv1; 5. ss11; 6. 14 mm.

Mitogenomes were generated using traditional Sanger sequencing at the Limnological Institute and sequencing-by-synthesis on Genome Analyzer IIx (Illumina, Inc., San Diego, CA, USA) at the ZAO Genoanalitica (Moscow, Russia). Sanger reads were trimmed and aligned with Bioedit 7.0.0 (Hall 2005), and Genome Analyzer reads were assembled using CLC Genomics Workbench 12.0 (QIAGEN, Aarhus, Denmark) on the HPC-cluster ‘Akademik V.M. Matrosov’ of Irkutsk Supercomputer Center SB RAS (http://hpc.icc.ru. . . . . . . . . . ). The mitogenomes of *Coregonus clupeaformis* and *Coregonus lavaretus* (Jacobsen et al. 2012) were served as reference sequences and used for phylogenetic reconstructions together with available mitogenomes of some other coregonids. A multiple alignment was conducted with ClustalW implementation in MEGA version 7 (Kumar et al. 2016) and validated by eye. Annotation pipeline MitoAnnotator (Iwasaki et al. 2013) was used for annotation of mitogenome sequences.

MEGA 7 (Kumar et al. 2016) was used to select the optimum nucleotide substitution model and conduct a maximum-likelihood phylogenetic analysis (Tamura et al. 2013; Figure 1). Minimum evolution and neighbor-joining trees resulted in the same tree topology as the maximum-likelihood tree. Phylogenetic analyses (Figure 1) confirmed interspecies relationships reported for Baikal coregonids based on Cytb mtDNA (Sukhanova et al. 2012). Comparable genetic distances (Figure 1) indicate a similar age between three lineages of interest: Baikal endemics, the North American lake whitefish, and the European whitefish.

**Figure 1.**
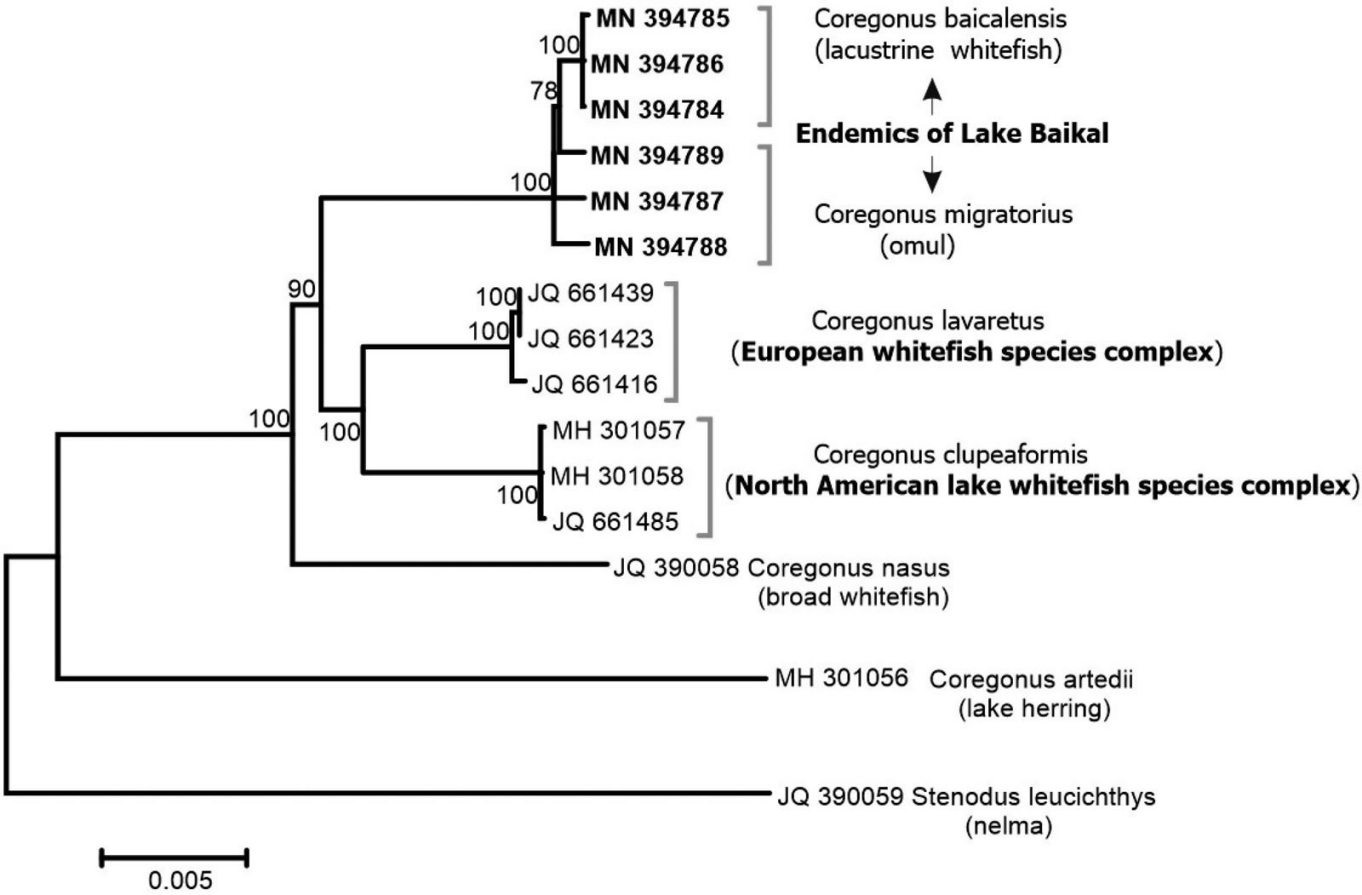
Interspecific phylogeny inferred under the maximum-likelihood (GTR +  G+I) optimality criterion (Nei and Kumar 2000). Support values represent the proportion of 500 bootstrap replicates in which the associated taxa clustered together. Evolutionary analyses were conducted in MEGA6 (Tamura et al. 2013).
